# Hmgb1 inhibits Klotho expression and malignant phenotype in melanoma cells by activating NF-κB

**DOI:** 10.18632/oncotarget.12623

**Published:** 2016-10-13

**Authors:** Biao Xie, Ke Cao, Jinjin Li, Jia Chen, Jintian Tang, Xiang Chen, Kun Xia, Xiao Zhou, Yan Cheng, Jianda Zhou, Huiqing Xie

**Affiliations:** ^1^ Deptment of Plastic Surgery, Third Xiangya Hospital, Central South University, Changsha, Hunan 410013, China; ^2^ Department of Colorectal Surgery, Second Affiliated Hospital, Hunan University of Chinese Medicine, Changsha, Hunan 410005, China; ^3^ Department of Oncology, Third Xiangya Hospital, Central South University, Changsha, Hunan 410013, China; ^4^ Institute of Medical Physics and Engineering, Department of Engineering Physics, Tsinghua University, Beijing, 100084, China; ^5^ Department of Dermatology, Xiangya Hospital, Central South University, Changsha, Hunan 410008, China; ^6^ State Key Laboratory, Medical Genetic, Central South University, Changsha, Hunan 410008, China; ^7^ Department of Oncoplast & Reconstructure Surgery, Affiliated Tumor Hospital, Central South University, Changsha, Hunan 410013, China; ^8^ School of Pharmacy, Central South University, Changsha, Hunan 410013, China; ^9^ Department of Rehabilitation, Third Xiangya Hospital, Central South University, Changsha, Hunan 410013, China

**Keywords:** Hmgb1, NF-κB, melanoma, Klotho, insulin signaling

## Abstract

The molecular and cellular mechanisms behind the involvement of inflammation in melanoma have not been fully elucidated. In this study, knockdown of Hmgb1 expression increased apoptosis, reduced invasion and p-NF-κB expression, but increased Klotho protein level in melanoma tumor cells. The effect of Hmgb1 knockdown was overcome by LPS. Introduction of exogenous Hmgb1 significantly decreased apoptosis, increased invasion, elevated p-NF-κB, but lowered Klotho protein level in melanoma cells. The effect of exogenous Hmgb1 was agonized by NF-κB inhibitor CAPE. Hmgb1 knockdown activated, but exogenous Hmgb1 inactivated, p-IGF1R/p-PI3K p-85/p-Akt/p-mTOR signaling. Knockdown of *Klotho* gene expression significantly decreased apoptosis, increased invasion in melanoma cells, and inhibited xenograft A375 tumor growth. A significantly high percentage of cells stained positive for p-NF-κB, but negative for Klotho, in melanoma tissues compared to normal and benign skin tissues. The positive p-NF-κB and negative Klotho protein expression correlated with poor prognosis in melanoma patients. Multivariate analysis revealed an independent association between p-NF-κB / Klotho protein level and overall survival. In conclusion, Hmgb1 can inhibit *Klotho* gene expression and malignant phenotype in melanoma cells through activation of NF-κB signaling.

## INTRODUCTION

Melanoma is a highly malignant tumor that is associated with the deaths of 50,000 people worldwide each year [1]. Currently, the incidence of melanoma is increasing worldwide [2]. However, the molecular mechanisms driving melanoma carcinogenesis and progression have not been fully elucidated.

Many tumors develop as a result of inflammation, and many biological characteristics of tumors exhibit a close relationship with the biological processes of inflammation [3]. NF-κB is an important nuclear transcription factor involved in tumor inflammation and development [4]. NF-κB can stimulate the expression of various cytokines and chemokines involved in epithelial cell growth and activity [5]. We hypothesize that NF-κB may be involved in the malignant transformation of epithelial cells.

Extracellular Hmgb1 is a member of the damage-associated molecular patterns and exerts an important role in the startup and promotion of inflammation as a danger signal [6]. Hmgb1 is involved in tumorigenesis and development as a late mediator of inflammation [7]. Our previous studies demonstrated that Hmgb1 is highly expressed in melanoma tissues, and is a predictive factor for the poor prognosis of melanoma patients [8]. Both Hmgb1 and LPS, an immune system activation factor, can activate NF-κB and subsequently promote tumor development [9]. A previous study demonstrated that TNF-α can suppress the expression of the anti-aging gene *Klotho* through activation of NF-κB [10]. In addition, the *Klotho* gene is progressively lost in melanoma under an unknown mechanism [11]. We therefore hypothesized that inflammation-activated NF-κB may activate Hmgb1, which subsequently suppresses *Klotho* gene expression.

This study investigated the effects of Hmgb1 and LPS on *Klotho* gene expression in melanoma cells and their relationship with NF-κB signaling and the biological significance of inflammation-Klotho in the malignant phenotype of melanoma.

## RESULTS

### Knockdown of Hmgb1 increased tumor cell apoptosis and decreased invasion in melanoma cells

**Figure 1:**
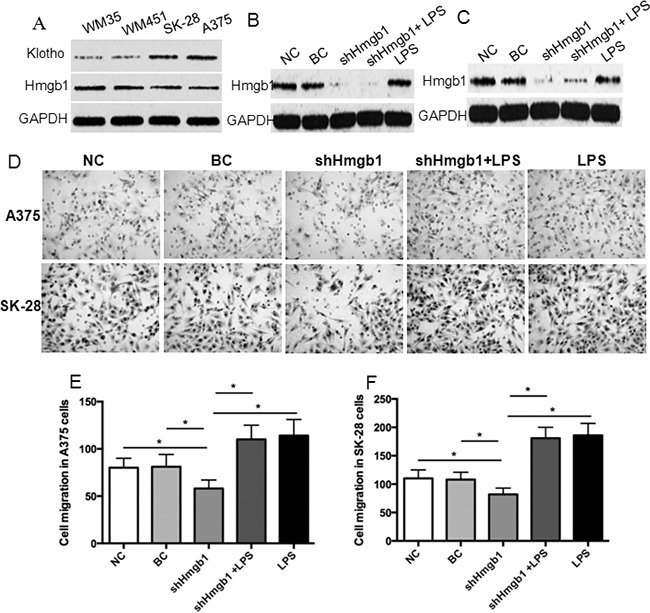
Knockdown of Hmgb1 expression decreases invasion in melanoma cells **A.** Western blot of Klotho and Hmgb1 protein expression in WM35, WM451, SK-28, A375 cells. Cells were transfected with or without pGFP-shHmgb1 and treated with or without LPS. NC: negative control. Cells were treated with transfection reagents. BC: blank control. Cells were transfected with blank vector. shHmgb1: cells were transfected with pGFP-shHmgb1 vector to express Hmgb1 shRNA. shHmgb1+LPS: cells were transfected with pGFP-shHmgb1 vector to express Hmgb1 shRNA and treated with LPS (10 ng/ml). LPS: cells were only treated with LPS (10 ng/ml). **B.** Evidence of the efficacy of Hmgb1 knockdown in A375 cells. **C.** Evidence of the efficacy of Hmgb1 knockdown in SK-28 cells. **D.** Representative photographs of cell invasion in A375and SK-28 cells. LPS: LPS alone. **E.** The number of cell invasion in A375 cells. **F.** The number of cell invasion in SK-28 cells. *P<0.001 between two groups. N=4.

**Figure 2:**
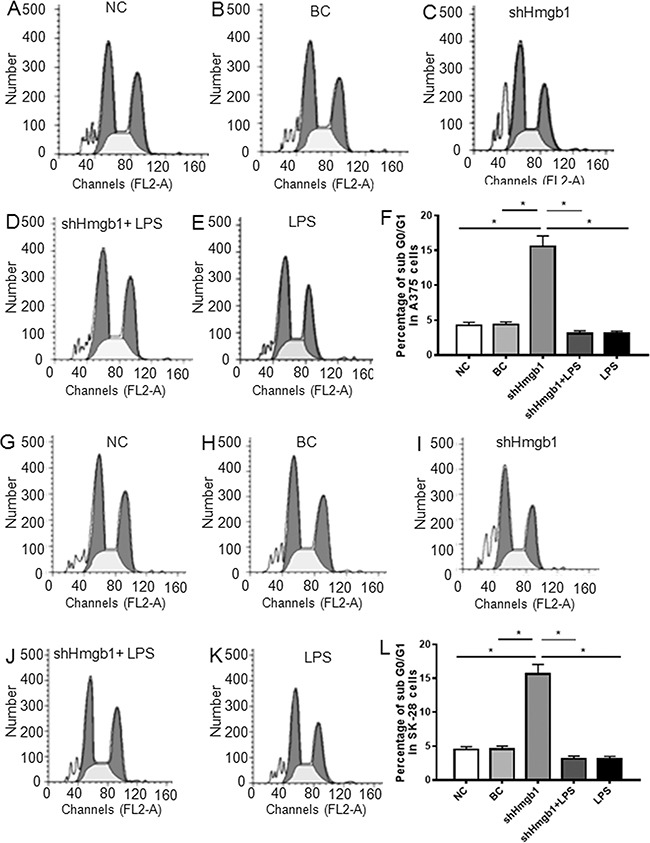
Knockdown of Hmgb1 expression increases sub G0/G1 cells in melanoma cells Cells were treated as described in Figure 1. NC: negative control. BC: blank control. shHmgb1: Expressing Hmgb1 shRNA. shHmgb1+LPS: pGFP-shHmgb1 + LPS. LPS: LPS alone. **A-E.** Representative flow cytometry assay of cell cycle in A375 cells. **F.** The percentage of sub G0/G1 cells in treated A375 cells. **G-K.** Representative flow cytometry assay of cell cycles in SK-28 cells. **L.** The percentage of sub G0/G1 cells in treated SK-28 cells. *P<0.05 between two groups. N=4.

**Figure 3:**
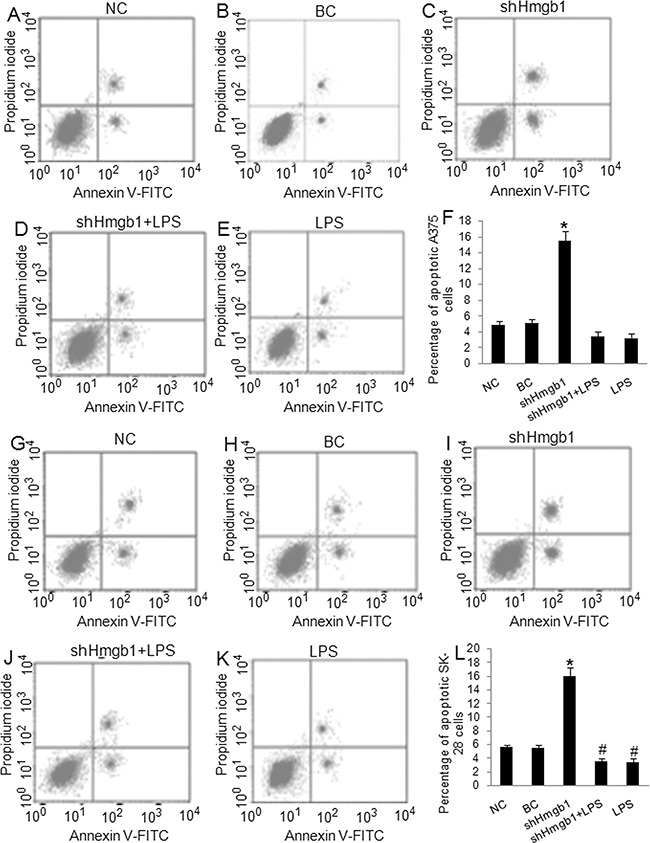
Knockdown of Hmgb1 expression increases apoptosis in melanoma cells Cells were treated as described in Figure 1. NC: negative control. BC: blank control. shHmgb1: Expressing Hmgb1 shRNA. shHmgb1+LPS: pGFP-shHmgb1 + LPS. LPS: LPS alone. **A-E.** Representative flow cytometry assay of apoptotic cells in A375 cells. The right up and down quadrant indicates the apoptotic cells. **F.** The percentage of apoptotic cells in treated A375 cells. **G-K.** Representative flow cytometry assay of apoptotic cells in SK-28 cells. The right up and down quadrant indicates the apoptotic cells. **L.** The percentage of apoptotic cells in treated SK-28 cells. *P<0.001 *vs.* other groups. ^#^P<0.05 *vs.* NC and BC group. N=4.

In this study, 4 melanoma cell lines were used to screen Klotho and Hmgb1 protein expression. Western blot showed that low Klotho protein expression and high Hmgb1 protein expression were detected in WM35 and WM451 cells, whereas high Klotho protein expression and low Hmgb1 protein expression were detected in SK-28 and A375 cells (Figure 1A). A375 and SK-28 cell lines with high Klotho protein expression were selected for further study. A pGFP-shHmgb1 vector was used to silence *Hmgb1* gene expression in A375 (Figure 1B) and SK-28 (Figure 1C) cells. 24 hrs after transfection, Western blot showed significant decrease in Hmgb1 protein. The Transwell assay in A375 (Figure 1D, 1E) and SK-28 (Figure 1D, 1F) cells showed that shHmgb1 transfection significantly reduced invasion, whereas LPS treatment significantly increased cell invasion compared to NC and BC cells (p<0.001). Invasion in cells treated with shHmgb1 transfection and LPS was significantly higher than that in the NC and BC cells (p<0.001). However, no significant differences in the invasion of cells were observed between treatments with shHmgb1 + LPS and LPS alone (p>0.05) (Figure 1D, 1E, 1F). Flow cytometry showed that shHmgb1 transfection significantly increased the percentage of sub G0/G1 in A375 (Figure 2A-2F) and SK-28 cells (Figure 2G-2L) (p<0.05). Also, LPS reversed the effect of shHmgb1 on cell cycle in two cell lines (P<0.05). shHmgb1 transfection significantly increased the percentage of cell apoptosis in A375 (Figure 3A-3F) and SK-28 cells (Figure 3G-3L) (p<0.001). Also, LPS reversed the effect of shHmgb1 on cell apoptosis in two cell lines (P<0.001).

### The effects of exogenous Hmgb1 in cell invasion, cell cycle, and apoptosis in A375 and SK-28 cells

**Figure 4:**
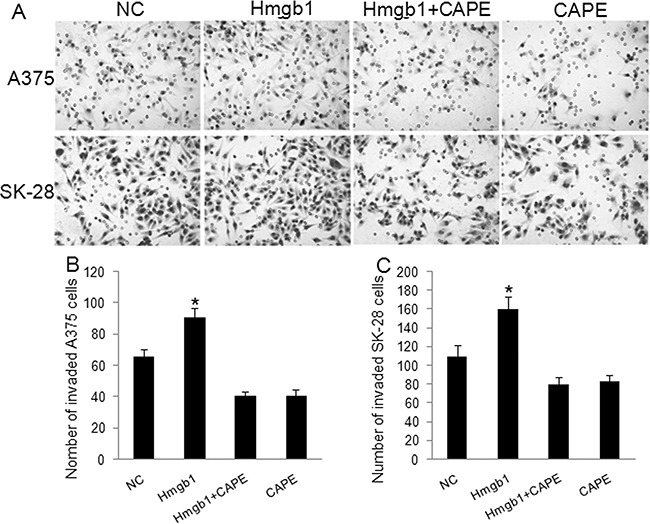
Exogenous Hmgb1 increased invasion in melanoma cells A375 and SK-28 cells were treated with Hmgb1 (0.1 μg/ml) with or without 100 μM of NF-κB inhibitor Caffeic Acid Phenethyl Ester (CAPE) for 48 hrs. NC: negative control. Cells were treated with PBS. Hmgb1: cells were treated with Hmgb1 protein (dissolved in PBS). Hmgb1+CAPE: cells were treated with Hmgb1 protein and CAPE. CAPE: cells were only treated with CAPE (dissolved in PBS). **A.** Representative photographs of cell invasion in A375 and SK-28 cells. **B.** The number of cell invasion in A375 cells. **C.** The number of cell invasion in SK-28 cells. ^*^P<0.05 *vs.* NC group, ^*^P<0.001 *vs.* other groups. N=4.

**Figure 5:**
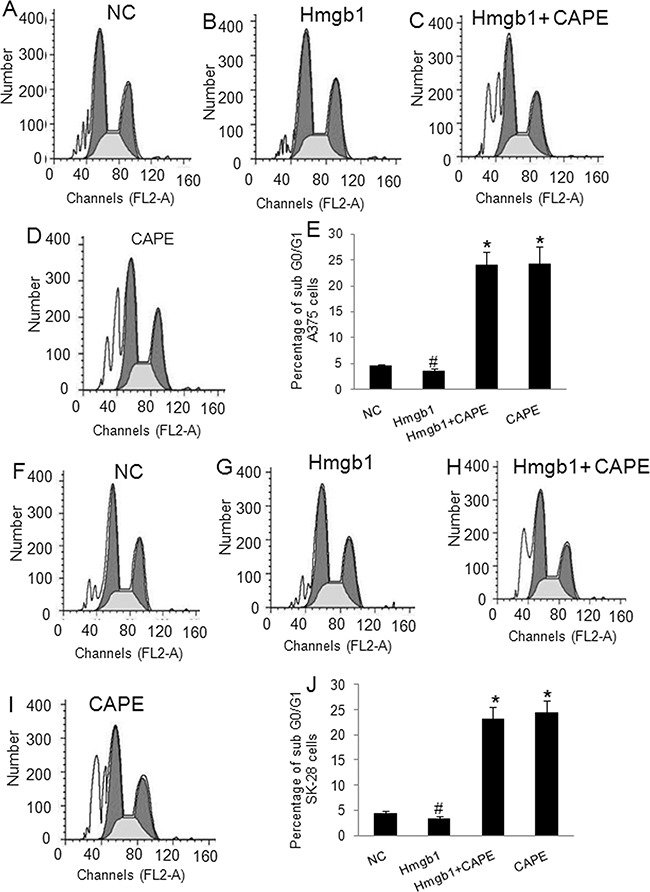
Exogenous Hmgb1 decreased sub G0/G1 cells in melanoma cells A375 and SK-28 cells were treated as described in Figure 4. NC: negative control. Hmgb1: Hmgb1 protein treatment. Hmgb1+CAPE: Hmgb1 protein and CAPE. CAPE: CAPE only. **A-D.** Representative flow cytometry assay of cell cycle in A375 cells. **E.** The percentage of sub G0/G1 cells in treated A375 cells. **F-I.** Representative flow cytometry assay of cell cycles in SK-28 cells. **J.** The percentage of sub G0/G1 cells in treated SK-28 cells. ^*^P<0.001 *vs.* other groups. ^#^p<0.05 *vs.* NC group. N=4.

**Figure 6:**
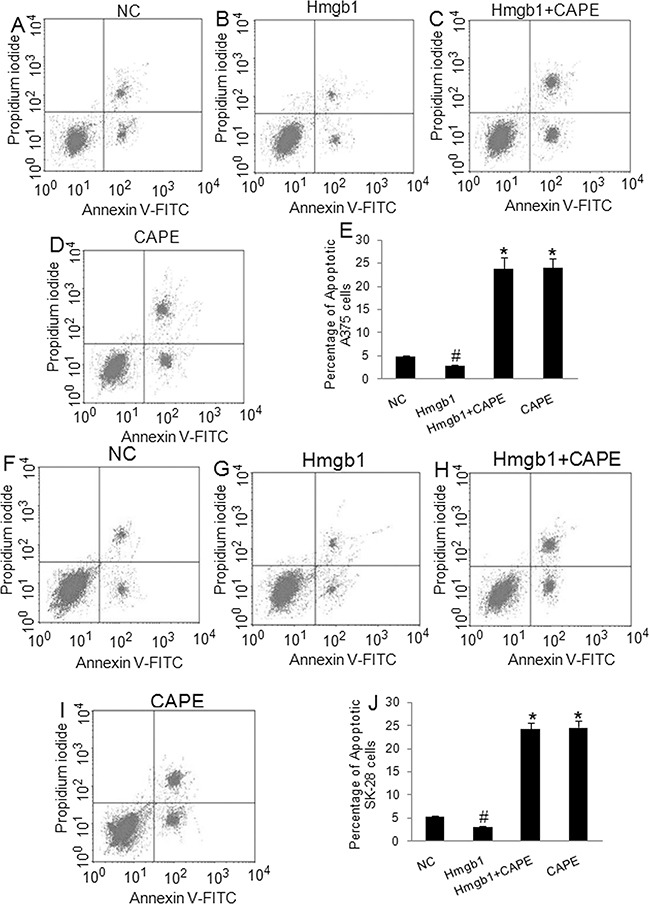
Exogenous Hmgb1 decreased apoptosis in melanoma cells A375 and SK-28 cells were treated as described in Figure 4. NC: negative control. Hmgb1: Hmgb1 protein treatment. Hmgb1+CAPE: Hmgb1 protein and CAPE. CAPE: CAPE only. **A-D.** Representative flow cytometry of apoptotic A375 cells. The right up and down quadrant indicates the apoptotic cells. **E.** The percentage of apoptotic A375 cells. **F-I.** Representative flow cytometry of apoptotic SK-28 cells. The right up and down quadrant indicates the apoptotic cells. **J.** The percentage of apoptotic SK-28 cells. ^*^P<0.001 *vs.* other groups. ^#^p<0.05 *vs.* NC group. N=4.

The A375 and SK-28 melanoma cells were treated with exogenous Hmgb1 at 0.01 μg/ml, 0.05 μg/ml, 0.1 μg/ml, and 0.5 μg/ml of Hmgb1 protein with or without 100 μM of NF-κB inhibitor CAPE for 24 and 48 hrs. 0.1 μg/ml and 0.5 μg/ml of Hmgb1 was similarly effective (data not shown). 0.1 μg/ml of Hmgb1 was selected for further experiments. Invasion test showed that exogenous Hmgb1 (0.1 μg/ml) treatment for 48 hrs significantly increased cell invasion in A375 (Figure 4A, 4B) and SK-28 cells (Figure 4A, 4C) (p<0.05). CAPE not only inhibited cell invasion, but also reversed the effects of Hmgb1 on cell invasion in both cell lines (p<0.001). Flow cytometry showed that Hmgb1 treatment for 48 hrs significantly decreased the percentage of sub G0/G1 A375 (Figure 5A-5E) and SK-28 cells (Figure 5F-5J) (p<0.05). CAPE not only increased the percentage of sub G0/G1 (p<0.001) cells, but also reversed the effects of Hmgb1 on the cell cycle in both cell lines (Figure 5E, 5J) (p<0.001). Exogenous Hmgb1 treatment significantly decreased the percentage of cell apoptosis in A375 (Figure 6A-6E) and SK-28 cells (Figure 6F-6J) (p<0.05). CAPE not only increased cell apoptosis (p<0.001), but also reversed the apoptotic effect of Hmgb1 in both cell lines (Figure 6E, 6J) (p<0.001).

### Hmgb1 / LPS can activate NF-κB signaling pathway and subsequently inhibit Klotho gene expression and its downstream signaling

**Figure 7:**
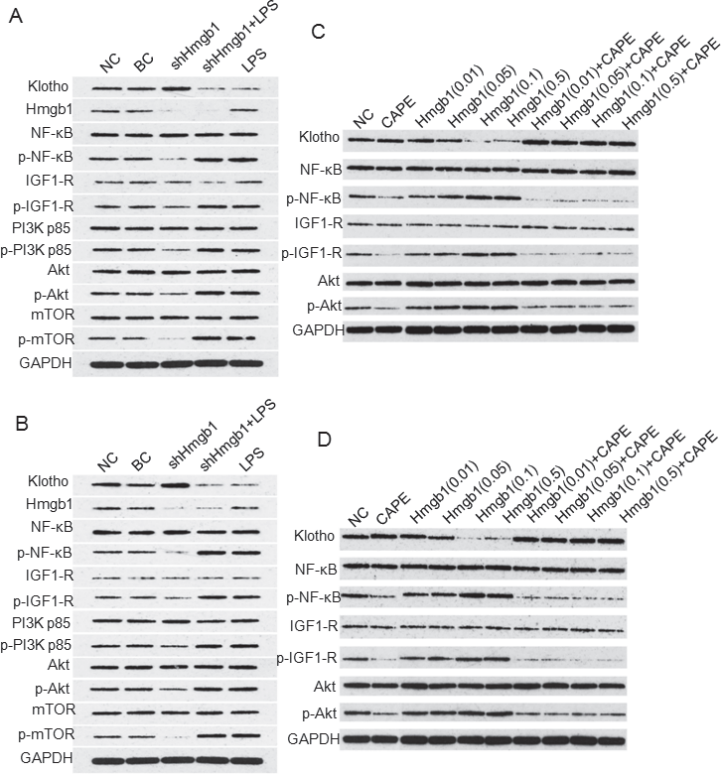
Western blot analysis of Klotho expression and NF-κB signaling Western blots for A375 **A.** and SK-28 **B.** cells treated with shHmgb1 and LPS. **C.** Western blots for A375 and **D.** for SK-28 cells treated with 0.01 μg/ml, 0.05 μg/ml, 0.1 μg/ml, and 0.5μg/ml of Hmgb1 with or without 100 μM of NF-κB inhibitor (CAPE) for 24 hrs.

The levels of p-NF-κB in shHmgb1 transfected A375 (Figure 7A) and SK-28 (Figure 7B) cells were significantly reduced compared to cells in the BC and NC control cells (p<0.001), suggesting that silencing of *Hmgb1* gene expression reduced NF-κB phosphorylation. Although the p-NF-κB levels in LPS treated and shHmgb1 + LPS treated A375 and SK-28 cells were significantly increased (p<0.001) compared to cells in the control (BC and NC) cells, no significance in p-NF-κB levels was observed between cells treated with shHmgb1 + LPS and LPS alone (p>0.05), suggesting that both the Hmgb1 and LPS can activate NF-κB (Figure 7A, 7B). We further found that Klotho protein expression in shHmgb1 treated A375 and SK-28 cells was significantly higher than that in control cells (p<0.001) (Figure 7A, 7B), suggesting that silencing of *Hmgb1* gene expression increased Klotho protein expression. In cells treated with LPS alone, no changes in Hmgb1 protein levels was observed, but the level of Klotho protein was significantly reduced compared to control cells (p<0.001). In A375 and SK-28 cells treated with shHmgb1 + LPS, Hmgb1 and Klotho protein expression was significantly reduced compared to control cells (p<0.001).

We further examined the expression of IGF-1R /PI3K / AKT / mTOR protein. In shHmgb1 treated A375 (Figure 7A) and SK-28 (Figure 7B) cells, the protein levels of p-IGF1-R, p-PI3K p85, p-Akt, and p-mTOR were significantly lower than that in control cells (p<0.001). In A375 (Figure 7A) and SK-28 (Figure 7B) cells treated with LPS, the levels of p-IGF1-R, p-PI3K p85, p-Akt, and p-mTOR were significantly higher than that in control cells (p <0.001). In cells treated with shHmgb1 + LPS or LPS alone, no significant differences in p-IGF1-R, p-PI3K p85, p-Akt, and p-mTOR levels were observed compared to control cells (p>0.05) (Figure 7A, 7B). Both the Hmgb1 and LPS can inhibit Klotho protein expression, which correlated with the activity of NF-κB signaling pathway.

In exogenous Hmgb1 treated cells, significant increas in p-NF-κB level, but decrease in Klotho protein expression, was observed in A375 (Figure 7C) and SK-28 (Figure 7D) melanoma cells. After treatment with p-NF-κB inhibitor CAPE, p-NF-κB level was significantly lowered, while the expression of Klotho protein was significantly increased (Figure 7C, 7D). When Klotho was highly expressed, p-IGF-1R / p-AKT levels were significantly reduced. When Klotho was lowly expressed, p-IGF-1R / p-AKT was significantly increased (Figure 7C, 7D). These findings suggest that Hmgb1 can activate NF-κB signaling pathway to decrease *Klotho* gene expression and the activity of downstream signaling.

### Knockdown of Klotho decreased melanoma tumor cell apoptosis and increased cell invasiveness

**Figure 8:**
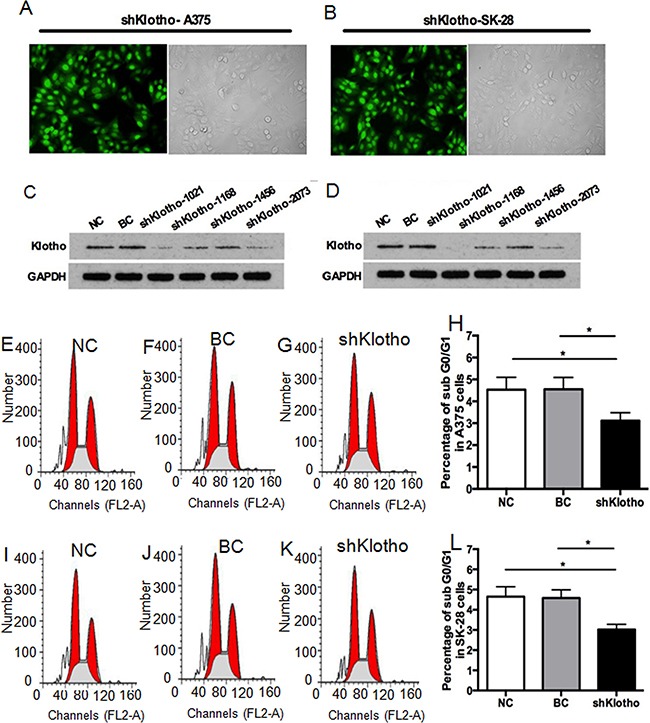
Knockdown of *klotho* gene expression decreased sub G0/G1 cells in treated melanoma cells **A.** Representative transfection efficacy of *Klotho* shRNA (shKlotho) in A375 cells. **B.** Representative transfection efficacy of shKlotho shRNA in SK-28 cells. **C.** Representative Western blot of Klotho and GAPDH protein expression with different Klotho shRNAs in A375 cells. **D.** Representative Western blot of Klotho and GAPDH protein expression in SK-28 cells. **E-G.** Representative flow cytometry assay of cell cycles in A375 cells. **H.** Percentage of sub G0/G1 A375 cells with different treatments. **I-K.** Representative flow cytometry assay of cell cycles in SK-28 cells. **L.** Percentage of sub G0/G1 cells in SK-28 cells. NC: negative control. Cells were treated with transfection reagents. BC: blank control. Cells were transfected with blank vector. shKlotho: cells were transfected with pGFP-shKlotho-1021 vector to express klotho shRNA. ^*^P<0.001 between two groups. N=4.

**Figure 9:**
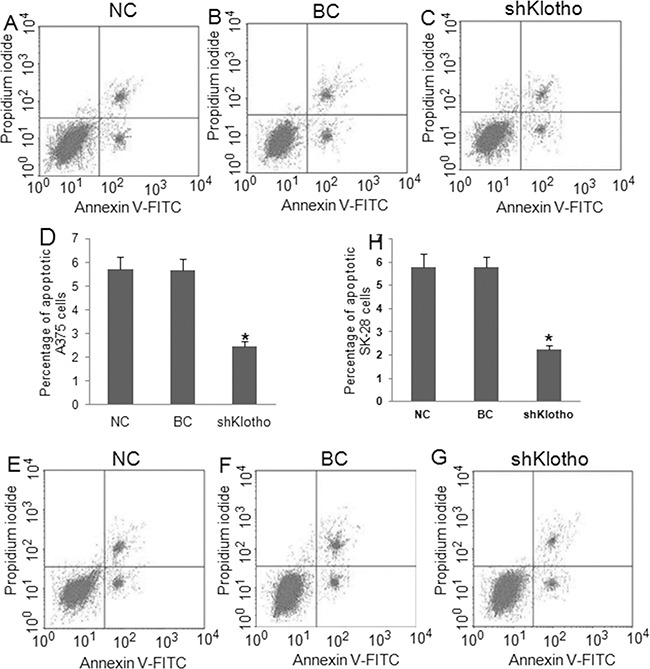
Knockdown of *Klotho* gene expression decreased apoptosis in melanoma cells Cells were treated as described in Figure 8. NC: negative control. BC: blank control. shklotho: klotho shRNA expression. **A-C.** Representative flow cytometry assay of apoptotic A375 cells with different treatments. The right up and down quadrant indicates the apoptotic cells. **D.** Percentage of apoptotic A375 cells. **E-G.** Representative flow cytometry assay of apoptotic SK-28 cells. The right up and down quadrant indicates the apoptotic cells. **H.** Percentage of apoptotic SK-28 cells. ^*^P<0.001 *vs.* other groups. N=4.

**Figure 10:**
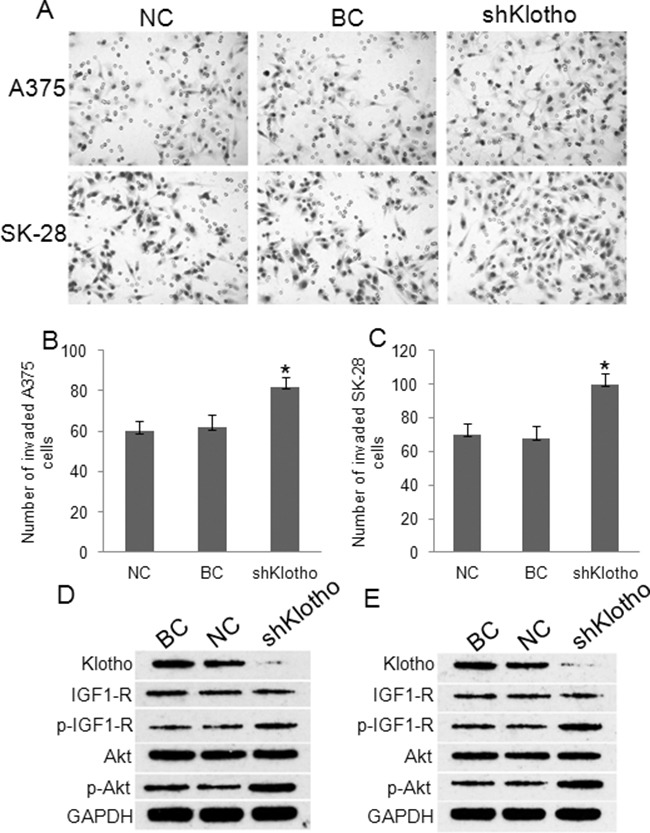
Knockdown of *Klotho* gene expression increased invasion and IGF-1R and Akt phosphorylation Cells were treated as described in Figure 8. NC: negative control. BC: blank control. shklotho: klotho shRNA expression. **A.** Representative photographs of cell invasion in A375 and SK-28 cells. **B.** The number of cell invasion in A375 cells. **C.** The number of cell invasion in SK-28 cells. ^*^P<0.001 *vs.* other two groups. N=4. **D.** Representative Western blots of protein expression in A375 cells. **E.** Representative Western blots of protein expression in SK-28 cells. Expression of Klotho shRNA obviously inhibited Klotho protein expression, but increased IGF-1R and Akt phosphorylation in A375 and SK-28 melanoma cells.

Transfection of Klotho shRNA expression vectors for 72 hrs established over 80% transfection efficacy in A375 (Figure 8A) and SK-28 (Figure 8B) cells. Western blot showed that transfection of shKlotho-1021 was most effect in inhibiting Klotho protein expression in A375 (Figure 8C) and SK-28 (Figure 8D) cells, and shKlotho-1021 was chosen for further tests. shKlotho transfection significantly decreased the percentage of sub G0/G1 in A375 (Figure 8E-8H) and SK-28 cells (Figure 8I-8L) (p<0.001). Flow cytometry showed that shKlotho transfection significantly decreased the percentage of cell apoptosis in A375 (Figure 9A-9D) and SK-28 cells (Figure 9E-9H) (p<0.001). Similarly, transfection of shKlotho vector significantly increased cell invasion in A375 (Figure 10A, 10B) and SK-28 cells (Figure 10A, 10C) (p<0.001). We further analyzed the signaling transduction in A375 (Figure 10D) and SK-28 (Figure 10E) cells. shKlotho transfection significantly inhibited Klotho protein expression. Also, shKlotho transfection significantly increased p-IGF1-R and p-Akt protein levels in A375 and SK-28 cells (p<0.001), but had no effect on the levels of IGF1-R and Akt protein in two cell lines (Figure 10D, 10E).

### Klotho knockdown stimulated xenograft tumor growth in mice

**Figure 11:**
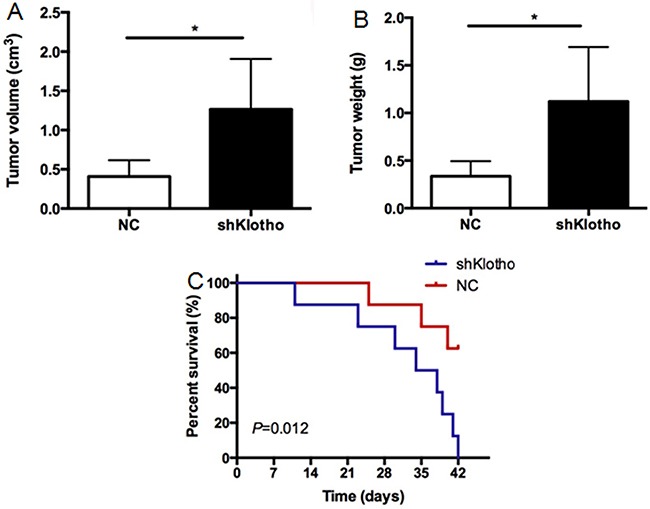
The effects of Klotho in xenograft melanoma tumor growth Xenograft A375 tumors were established as described in Methods. **A.** The final tumor volume of xenograft A375 tumors in mice. Mice were sacrificed at 42 days after inoculation. **B.** The weights of xenograft A375 tumors. **C.** The survival of mice carrying A375 tumors. N=8. ^*^P<0.001 between two groups.

We further validated the effect of *Klotho* gene knockdown on the growth of xenograft A375 tumors in mice. The shKlotho or scrambled control-shRNA (NC) transfected A375 cells were injected subcutaneously in nude mice. After 42 days, the mice were sacrificed. The average volume of the tumors in the shKlotho group was significantly higher than that in the control (NC) group (1.264±0.642 *vs.* 0.408±0.207cm^3^, Figure 11A, p<0.05). The average tumor weight in the shKlotho group was 1.121±0.572g, which was significantly higher than that in the control group (0.335±0.159 g) (Figure 11B, p<0.05). Kaplan-Meier analysis showed that the survival rate of mice in shKlotho group was lower than that in NC group (Figure 11C, p<0.05). These results suggest that down-regulation of *Klotho* gene expression significantly enhanced xenograft melanoma tumor growth.

### NF-κB activation and low Klotho protein expression correlated with poor prognosis of melanoma patients

**Figure 12:**
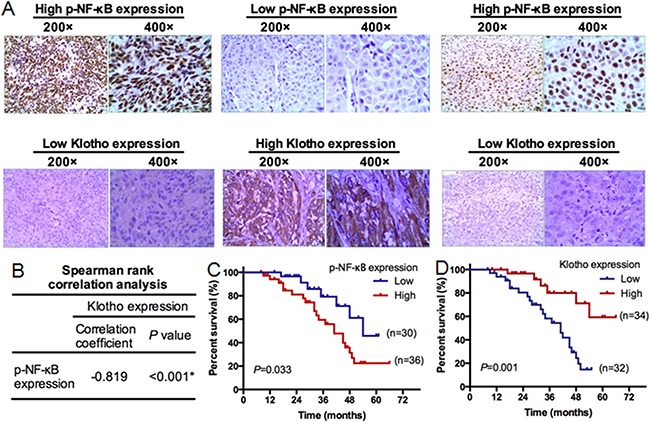
Klotho expression in human melanoma tissues and its clinical significance Sixty-six melanoma tumor tissues were subjected to immunohistochemical staining of p-NF-κB and Klotho protein. **A.** Representative immunohistochemical staining of p-NF-κB and Klotho expression. Left panels: high p-NF-κB expression, low Klotho expression (200x, 400x) in melanoma tissues. Middle panels: low p-NF-κB expression, high Klotho expression (200x, 400x) in melanoma tissues. Right panels: high p-NF-κB expression and low Klotho expression (200x, 400x) in melanoma tissues with ulceration. **B.** The spearman rank correlation analysis between the expression of Klotho and p-NF-κB. **C.** Kaplan-Meier analysis of survival in patients with high or low p-NF-κB levels. N=66. **D.** Kaplan-Meier analysis of survival in patients with high or low Klotho expression. N=66.

Immunohistochemistry showed that p-NF-κB protein staining was mainly located in the nucleus, while Klotho protein staining was mainly located in the cytoplasm and cell membrane (Figure 12A). Positive p-NF-κB protein staining was observed in 68% of melanoma tissues, which was significantly higher than that in the black nevus carcinoma and peritumoral tissues (23%). Positive Klotho protein staining was observed in 45% of melanoma tissues, which was significantly lower than that in black nevus carcinoma and peritumoral tissues (85%). p-NF-κB (Table 1) and Klotho (Table 2) protein expression in melanoma tissues significantly correlated with the clinical stage (P<0.05). The result of the spearman rank correlation analysis revealed that the expression of Klotho protein negatively correlated with p-NF-κB protein (Figure 12B, r = 0.819, P < 0.001). Kaplan-Meier analysis showed that the prognosis of patients with high p-NF-κB protein levels (++, +++) was significantly poor than patients with low p-NF-κB levels (-, +) (Figure 12C). In contrast, low Klotho protein levels (-, +) correlated with a poor prognosis (Figure 12D). Multivariate analysis based on Cox models was also carried out to further explore the prognostic significance of p-NF-κB (Table 3) and Klotho (Table 4) protein. The results revealed that p-NF-κB and Klotho protein levels were independent predictors of poor overall survival, indicating that high p-NF-κB or low Klotho protein levels were significantly associated with poor overall survival in melanoma patients (p<0.05).

**Table 1:** Correlation between the expression of p-NF-κB and clinicopathological parameters in melanoma patients

Characteristics		p-NF-κB expression				P value
		−	+	++	+++	
Age						
	<50	8 (19.5%)	12 (29.3%)	10 (24.4%)	11 (26.8%)	0.609
	≥50	2 (8.0%)	8 (32.0%)	6 (24.0%)	9 (36.0%)	
Gender						
	Male	3 (10.0%)	6 (20.0%)	11 (36.7%)	10 (33.3%)	0.087
	Female	7 (19.4%)	14 (38.9%)	5 (13.9%)	10 (27.8%)	
Ulcer history						
	Yes	8 (20.0%)	11 (27.5%)	9 (22.5%)	12 (30.0%)	0.579
	No	2 (7.7%)	9 (34.6%)	7 (26.9%)	8 (30.8%)	
Position						
	Limbs	8 (17.8%)	11 (24.4%)	12 (26.7%)	14 (31.1%)	0.454
	Truncus	2 (9.5%)	9 (42.9%)	4 (19.0%)	6 (28.6%)	
Clinical stage						
	I-II	7 (21.9%)	9 (28.1%)	11 (34.4%)	5 (15.6%)	0.029*
	III-IV	3 (8.8%)	11 (32.4%)	5 (14.7%)	15 (44.1%)	
Clark level						
	I-IV	7 (21.2%)	10 (30.3%)	8 (24.2%)	8 (24.2%)	0.494
	V	3 (9.1%)	10 (30.3%)	8 (24.2%)	12 (36.4%)	
Breslow thickness						
	<4mm	6 (18.2%)	13 (39.4%)	5 (15.2%)	9 (27.3%)	0.199
	≥4mm	4 (12.1%)	7 (21.2%)	11 (33.3%)	11 (33.3%)	

* P<0.05

**Table 2:** Correlation between the expression of Klotho and clinicopathological parameters in melanoma patients

Characteristics		Klotho expression				P value
		−	+	++	+++	
Age						
	<50	7 (17.1%)	11 (26.8%)	11 (26.8%)	12 (29.3%)	0.317
	≥50	5 (20.0%)	11 (44.0%)	6 (24.0%)	3 (12.0%)	
Gender						
	Male	8 (26.7%)	9 (30.0%)	10 (33.3%)	3 (10.0%)	0.057
	Female	4 (11.1%)	13 (36.1%)	7 (19.4%)	12 (33.3%)	
Ulcer history						
	Yes	4 (10.0%)	15 (37.5%)	11 (27.5%)	10 (25.0%)	0.202
	No	8 (30.8%)	7 (26.9%)	6 (23.1%)	5 (19.2%)	
Position						
	Limbs	7 (15.6%)	17 (37.8%)	9 (20.0%)	12 (26.7%)	0.245
	Truncus	5 (23.8%)	5 (23.8%)	8 (38.1%)	3 (14.3%)	
Clinical stage						
	I-II	5 (15.6%)	11 (34.4%)	4 (12.5%)	12 (37.5%)	0.015*
	III-IV	7 (20.6%)	11 (32.4%)	13 (38.2%)	3 (8.8%)	
Clark level						
	I-IV	5 (15.2%)	8 (24.2%)	12 (36.4%)	8 (24.2%)	0.178
	V	7 (21.2%)	14 (42.4%)	5 (15.2%)	7 (21.2%)	
Breslow thickness						
	<4mm	4 (12.1%)	11 (33.3%)	8 (24.2%)	10 (30.3%)	0.383
	≥4mm	8 (24.2%)	11 (33.3%)	9 (27.3%)	5 (15.2%)	

* P<0.05

**Table 3:** Multivariate analyses for overall survival by Cox regression model

	P value	Exp(B)	RR 95% CI	
			Lower	Upper
Age	0.102	2.192	0.856	5.615
Gender	0.55	0.763	0.315	1.851
Ulcer history	0.368	0.643	0.247	1.68
Position	0.908	0.946	0.368	2.429
Clinical stage	0.001*	5.484	2.033	14.792
Clark level	0.011*	3.001	1.285	7.012
Breslow thickness	0.222	0.576	0.237	1.398
p-NF-κB	0.037*	2.887	1.066	7.82

* P<0.05

**Table 4:** Multivariate analyses for overall survival by Cox regression model

	P value	Exp(B)	RR 95% CI	
			Lower	Upper
Age	0.372	1.567	0.584	4.206
Gender	0.844	0.918	0.392	2.151
Ulcer history	0.551	0.74	0.274	1.994
Position	0.856	1.095	0.413	2.9
Clinical stage	0.001*	5.326	1.986	14.281
Clark level	0.027*	2.706	1.121	6.533
Breslow thickness	0.351	0.661	0.277	1.578
Klotho	0.022*	0.283	0.096	0.836

* P<0.05

## DISCUSSION

Inflammation and inflammatory cytokines have been associated with tumor occurrence and development. This study showed that silencing Hmgb1 expression significantly increased the percentage of G0/G1 cells and apoptosis in melanoma cells, and reduced tumor cell invasion. In contrast, administration of exogenous Hmgb1 decreased apoptosis and the percentage of G0/G1 cells, and increased cell invasion and NF-κB phosphorylation in the melanoma cells. These findings suggest that Hmgb1 is involved in the development of melanoma. We also found that LPS can stimulate the invasion of melanoma cells, reduce apoptosis, and activate NF-κB. LPS and Hmgb1 activated NF-κB and enhanced tumor cell proliferation. Our study suggests a pathway of inflammation-activated NF-κB-Hmgb1-Klotho in the cell survival, proliferation, and tumor growth of melanoma.

NF-κB is an important nuclear transcription factor associated with tumor inflammation [12]. After activation, NF-κB promotes the production of cytokines, chemokines, growth factors, and apoptotic proteins [13]. This study found that phospho-NF-κB levels were significantly higher in melanoma tissues than in black nevus and peritumoral tissues. Melanoma patients with high phospho-NF-κB levels had poor prognosis. Experiments with two melanoma cell lines showed that both Hmgb1 protein and LPS treatment increased the malignant phenotypes of melanoma cells via activation of NF-κB. Moreover, LPS can totally overcome the phenotypes and the decrease in Hmgb1 protein expression caused by Hmgb1 silencing. In contrast, treatment with NF-κB inhibitor CAPE can reverse the phenotypes caused by exogenous Hmgb1. These findings suggest that inflammation-activated NF-κB can stimulate Hmgb1 expression and its biological activities.

Previous reports implicate an association between inflammation and *Klotho* gene expression. For example, the inflammatory mediator TNF-α could significantly inhibit *Klotho* gene expression in renal epithelial cells [10] and fat cells [14]. This study found that Klotho protein expression was significantly lower in melanoma tissues than that in peritumoral tissues and black nevus tissues. The low Klotho protein expression correlated with high percentage of positive p-NF-κB staining in melanoma tissues. Low Klotho protein expression significantly correlated with poor prognosis of patients with melanoma. We also found that when NF-κB signaling was inhibited, Klotho protein expression was significantly increased. In contrast, when NF-κB signaling was activated, Klotho protein expression was significantly decreased. In short, we found that *Klotho* gene expression in melanoma cells is inhibited by inflammatory factors NF-κB.

Our previously studies found that Klotho protein inhibited the activation of IRS-1 / PI3K / Akt / mTOR and ERK / p70s6k and downstream signaling pathways in gastric cancer and liver cancer cells, mainly through the inhibition of IGF-1R phosphorylation [15, 16]. This study found that after silencing *Klotho* gene, p-IGF-1R / p-AKT levels and cell invasion were significantly increased, whereas tumor cell apoptosis was significantly decreased. At the same time, the p-IGF-1R / p-PI3K / p-Akt / p-mTOR's levels were also increased or decreased with the activation or inhibitor of NF-κB. These findings indicate that Klotho may be an intermediate factor of NF-κB and insulin signaling pathways, and is involved in the malignant phenotypes of melanoma. We further validated that down-regulation of Klotho protein expression stimulated growth of xenograft A375 tumors in mice.

In conclusion, our study first proposed that inflammation inhibits *Klotho* gene expression in melanoma cells through activation of the inflammatory mediators Hmgb1 and NF-κB signal pathway.

## MATERIALS AND METHODS

### Samples

74 melanoma tissues were collected from the Second Xiangya Hospital and Third Xiangya Hospital, Central South University between 2002 and 2010 at the Department of Pathology. After passing the exclusion criteria including insufficient patient information and tumor material, 66 samples were finally collected for this study. 20 black nevi and peritumoral tissues were collected as control at the same stage. Melanoma was diagnosed by HE staining, HMB45 and S-100 protein immunohistochemical staining by three pathologists (double blind method). None of the 66 melanoma patients received chemotherapy and radiotherapy before surgery. Among the 66 melanoma patients, 30 were male and 36 were female with ages ranging from 28 to 65 years old. All specimens were fixed in 10% neutral formalin and embedded in paraffin. Tissues were sectioned at a thickness of 4 μm and stored at room temperature. The follow-up was performed by mail and telephone. The study was compliant with ethical standards.

### Immunohistochemistry

Sections were deparaffinized with xylene followed by hydration in gradient ethanol and 3% hydrogen peroxide solution to eliminate endogenous peroxidase. After washing with 1xPBS buffer (pH7.4), antigen retrieval was performed by heating the sections in citric acid buffer (pH6.0) in a microwave oven at 750W for 12 min, cooling for 3 minutes, and heating at 750W for 12 minutes again, followed by cooling naturally to room temperature. After blocking at room temperature for 30 min, sections were incubated with mouse anti-human p-NF-κB and Klotho antibodies overnight at 4°C. After washing with 1xPBS buffer, sections were incubated with HRP-conjugated goat anti-mouse secondary antibody (Beijing Zhongshan company, China) at 37°C for 60 min. After incubation with substrate (S-A solution in SP kit, Beijing Zhongshan Company) for 30 min at room temperature, color development was performed with DAB solution and counterstained with hematoxylin, followed by dehydration in gradient ethanol. Positive tissue sections were used as positive controls. Primary antibody was replaced with PBS as a negative control. Brown staining in the cytoplasm, membrane, and nucleus was justified as positive. 10 high-power fields were observed for each slice. The results are divided into: <5% (-), 6% to 30% (+), 3l % to 60% (++),> 60% (+++) according to the percentage of positive cells and the degree of staining intensity of cells. A case was defined as high expression when more than 31% (++ to +++) of cells were positively stained, while a case was defined as low expression when less than 30%, and the (- to +) of cells were positive.

### Cell culture

Human melanoma cell lines WM35, WM451, SK-28, and A375 were purchased from the Cell Bank of Shanghai Institute. Cells were cultured in RPMI 1640 (GIBCO) containing 10% fetal bovine serum at 37 °C, 5% CO_2_.

### Apoptosis and cell cycle analysis

To detect apoptosis, cells were digested with 0.25% EDTA-free trypsin and collected by centrifugation at 2,000 rpm for 5 min. After washing with 1 × PBS, 1 ~ 5 × 10^5^ cells were suspended in 500 μL of binding buffer. 5 μL of annexin V-FITC was then added, followed by the addition of 5 μL of propidium iodide, and incubation at room temperature for 10 min in the dark room. Apoptotic cells were sorted by flow cytometry.

To perform cell cycle analysis, cells were collected as described above and fixed in pre-cooled 75% ethanol overnight at 4°C. After washing with 1×PBS (1000rpm, 5min), cells were resuspended in a solution of 800 μl of 1×PBS containing 1% BSA. After adding 100 μl of PI dye (3.8 × 10^-2^ M sodium citrate, pH 7.0) and 100 μl of RNase A (10 mg / ml), cells were incubated at 37°C for 30 min in the dark. Cell cycles were analyzed by flow cytometry.

### Western blot analysis

Cells were lysated and total protein was extracted. Western blot was performed as previously described [17]. Anti-Klotho, anti-p-IGF-IR, anti-IGF-IR, anti-p-AKT1, and anti-GAPDH antibodies were purchased from Santa Cruz biotechnology (Santa Cruz, CA, USA). Anti-AKT antibody, anti-p-PI3K, and anti-p-mTOR antibodies were purchased from Cell Signaling Technology (Danvers, MA, US).

### Cell invasion assay

After digestion with 0.25% EDTA-free trypsin, cells were washed once with 1×PBS and suspended in RPMI 1640 medium containing 1% BSA at 5 × 10^4^/ml. 1 ml FBS-containing medium was added to 6-well plates and 2 ml cell suspension was added into the Transwell chamber. Cells were cultured at designated time points and membrane was removed from the lower chamber. Matrigel was wiped with a cotton swab and cells were fixed with 95% alcohol for 10 min followed by hematoxylin staining for 10 minutes. Cells were counted under inverted microscope.

### Cell transfection experiments

5 × 10^4^ cells in 2 mL FBS-containing medium were seeded in each well of 6-well plates 24 hrs before transfection. Cells at 70% confluency were transfected with Hmgb1-shRNA and Klotho-shRNA expression vector (2 μg/ml) using Lipofectamine ™ 2000 reagent by following the user manual. The Hmgb1-shRNA expression vector was constructed by directionally cloning of a mini gene containing a hairpin of shRNA sequence (5’-GCAAGTATTCGGTGCTATATA-3’) of Hmgb1 gene with a 9-mer loop and 3’ terminal uridine tract into the pGPU6/GFP/Neo vector at Bam HI and Bbs I sites. The produced vector was called pGFP-shHmgb1. Klotho-shRNA1 (5’-GCCAATTGGAATCTCCCAACC-3’), Klotho-shRNA2 (5’-GCCAGGACAAGATGTTGTTGC-3’), Klotho-shRNA3 (5’-GAGCCGTATACAAGGAATATG-3’), and Klotho-shRNA4 (5’-CCGAGAGCATGAAGAATAACC-3’) expression vector was constructed as described above and called pGFP-shKlotho1, pGFP-shKlotho2, pGFP-shKlotho3, and pGFP-shKlotho4. NF-κB activator (LPS, 1μg / ml), NF-κB inhibitor (Caffeic Acid Phenethyl Ester, called CAPE, 200 μM), and HMGB1 (0.01 μg / ml, 0.05 μg / ml, 0.1 μg / ml, and 0.5 μg / ml) were added.

### Nude mouse xenograft studies

Sixteen male BALB/c-nu/nu (aged 4-6 weeks) mice were purchased from the animal laboratory of the Third Xiangya Hospital, Central South University. To explore the role of Klotho in melanoma cells, xenograft tumors were grown in nude mice. The shKlotho or scrambled control-shRNA transfected A375 cells (2×10^6^) were injected subcutaneously in the ventral trunk of mice. Nude mice were sacrificed at 42 days after tumor implantation. Tumor volume, weight, and the survival rates of mice were measured. The experimental procedures were approved by the Ethics Committee of the Faculty of Experimental Animals, Central South University.

### Statistic analysis

Statistical analysis was performed using SPSS13.0 statistical software. The count data were analyzed using χ^2^ test, while the measurement data were analyzed using independent sample t-test. The comparison of count data for multiple samples was performed using rows × columns χ^2^ test. Spearman rank correlation analysis was used to detect the relationship between p-NF-κB and Klotho protein expression. Survival was analyzed by Kaplan-Meier method and the Log-rank test. Multivariate survival analysis was performed using the Cox multivariate analysis model. ^*^P<0.05 was considered statistically significant.
